# Nurturing by nutrition: On the future of gut microbiota management strategies for autoimmune disease

**DOI:** 10.3389/fnut.2022.1107016

**Published:** 2023-01-12

**Authors:** Olaf F. A. Larsen

**Affiliations:** Athena Institute, Vrije Universiteit Amsterdam, Amsterdam, Netherlands

**Keywords:** gut microbiota, old friends, autoimmune, probiotics, nutrition, One Health, fecal microbiota transfer (FMT)

## Abstract

The incidence of autoimmune disease continues to rise, which urges for new prevention and treatment modalities. The composition of the gut microbiota is associated with both susceptibility and progression of disease. Nutrition significantly shapes the gut microbial composition, and poses as such a modality for both prevention and treatment/adjuvant therapy. At very young age, nutritional intervention targeting the gut microbiota is still possible within a one-size-fits all regime, accompanied by a relatively high effect size. As ageing results in higher interindividual variation induced by cumulative exposome factors, a more personalized approach is needed, having a higher effect size than that of current nutritional intervention. As such, supplementation of microbial consortia consisting of keystone taxa and microbial guilds that are involved in the pathophysiology seem a promising direction to lower the burden of autoimmune disease.

## Introduction

The incidence of autoimmune disease is on the rise, as has been shown for the last half the 20th century in a landmark paper by Bach (1). Recently it was shown that this rising trend continues in the 21st century as well (2). As the treatment of autoimmune disorders remains difficult and relatively in its infancy, alternative treatment and prevention modalities are urgently needed (3). The pathogenesis of autoimmune disorders is known to be associated with both genetic and environmental factors (4). The composition of the gut microbiota is strongly determined by environmental factors (5), and it has been shown that a dysbiosis is associated to the disease status (6). As such, adequate prevention and treatment of autoimmune disorders call for a One Health approach, also involving the (targeted) stimulation and supplementation of beneficial microbes (7). As the gut microbiota composition is intimately related to dietary patterns (8), nutrition may provide a useful strategy as such. In this perspective, possible future directions toward gut microbiota management using dietary intervention to reduce the burden of autoimmune disease will be discussed.

## Autoimmune disease: Old friends and dysbiosis

The current understanding of the aforementioned epidemiologic rise in autoimmune disorders is that it is at least partly being caused by an insufficient exposure to beneficial microbes at early age (9). This theory is nowadays known as the “old friends hypothesis” (10), which claims that this insufficient exposure leads to a not fully developed immune system. As such, exposure at older age to relatively harmless microorganisms, but also to “self” antigens, may lead to an excessive immune reaction, which can be either inflammatory or allergic (11). The old friends hypothesis is a refinement of the “hygiene hypothesis,” which was proposed earlier (12), stating that early childhood infections could prevent atopic disease. As such, excessive hygiene could lead to diminished exposure to infections, leading to a reduced Th1 activity, which was assumed to be accompanied by a “compensating” increase in Th2 response leading to allergic disease (13). However, later studies showed results that were in conflict with the proposed mechanism underlying the hygiene hypothesis, as inflammatory bowel disease and autoimmune disorders were recognized to be associated with an increase in Th1, instead of the expected decrease (13). The theory was refuted, with a plead for “targeted hygiene” that maximizes protection against pathogens while simultaneously stimulating the exposure to harmless microorganisms (14). Nowadays, it is proposed that exposure to non-pathogenic microorganisms evokes a dampening response by the immune system, represented by regulatory T cells (Treg). Indeed, it has been shown that children that are being raised in a farm-like environment, and as such more exposed to “old friends,” develop less autoimmune type disorders like hay fever or asthma as compared to children that are being raised in a more urban environment (15). Intervention with children in daycare settings also showed that targeted intervention by the introduction of “biodiversity elements,” leading to a higher exposure to soil microbiota, was related to a decreased inflammatory status, as indicated by an increased IL-10:IL-17 ratio (16).

Patients suffering from autoimmune disorders, like those having rheumatoid arthritis, systemic lupus erythematosus or spondyloarthritis are shown to have an altered gut microbiota (17). In general, patients present a compromised gut barrier, leading to a leaky gut, which gives rise to an aberrant translocation of microbial taxa ultimately resulting in an increased inflammatory status (17). Although the presence or absence of particular microbial taxa do show associations with disease susceptibility and progression, no “microbial blueprints” are currently being identified for autoimmune disorders, which is also the case for all other non-communicable and infectious disease (except for, obviously, a *Clostridium difficile* infection). The identification of such generic microbial blueprints may very well never be possible, as it seems unlikely that “the” healthy microbiota will never be found due to the large interindividual variation, although certain microbial features of a healthy microbiota have recently been identified (5). Moreover, next to the large interindividual variation caused by factors like diet, medication and mode of delivery, the gut microbiota composition is also shaped by living arrangements (urban or rural) and geographical location (18). As such, there are differences found in gut microbiota composition between children from rural Africa and Europe (19).

## Discussion: Targeting the gut microbiota for autoimmune disease: From current status to future perspectives

Modulating the gut microbiota at very early age may be a promising strategy to reduce the burden of autoimmune disease, because it is in the realm of prevention. “Natural interventions” to program the health status at very early age could well be the most preferred interventional modality. Indeed, breastfeeding was associated by a reduction in numerous autoimmune disorders like celiac disease and multiple sclerosis (20). Breastmilk contains, among others, oligosaccharides that serve as natural prebiotics, as well as microorganisms like lactobacilli and bifidobacteria (21, 22). Infant nutrition that provides an alternative in case breastfeeding is not possible is therefore also a topic of intense study (23).

Early intervention with the probiotic strain *Lactobacillus Rhamnosus* GG to reduce the probability to develop atopic eczema and asthma revealed conflicting results so far (24, 25). While in the Finish study conducted by Kalliomäki positive results were obtained, no effect could be observed in the American counterpart conducted by Cabana. A reason for this could be that the intervention studies were performed in either Finland or in the USA, which may be associated to different baseline microbiota profiles and/or (epi)genetic programming resulting in different response rates to the probiotic intervention. Nevertheless, early age steering of the gut microbiota composition may provide an attractive modality to prevent autoimmune disease, as the gut microbiota is not fully developed yet and, especially in the first 3 years of life, still accepts microorganisms for permanent colonization (26). Indeed, as mentioned before, natural exposure to old friends modulating the gut microbiota at very young age resulted in a lowering of the probability to develop autoimmune disease (15).

Children at very early age still have, inherently, a relatively low exposure to external environmental factors, the so-called exposome. As a consequence, the interindividual variation between children at very early age is also relatively low. Their exposome will be predominantly determined by a “baseline exposome,” which is composed of genetic and epigenetic factors that were already present at birth. Consequently, the number needed to treat for a “one-size-fits-all” nutritional intervention (like a standard single- or multi-strain probiotic preparation) will be relatively low when starting at young age [with the number needed to treat being the number of patients who must be treated in order to prevent one adverse event, which is the equivalent of the reciprocal of the absolute risk reduction (27)]. Ageing is inherently associated with an increased exposure to all kind of environmental factors like dietary patterns, medication like antibiotics, emotional stress, and pollution. The effects of these factors, determining the total cumulative exposome, which is inherently unique for every individual, perturbs the composition of the gut microbiota (either positively or negatively), leading to an inherently larger interindividual variation with increasing age. As such, the number needed to treat for a one-size-fits all nutritional intervention will increase with increasing age.

Early life exposure to exposome factors that modify the infant gut microbiota are known to have effects on the health status at later age (28). Hence, it can therefore be expected that the effect size of early life interventions targeting the gut microbiota, that can be realized by nutritional intervention like using pre- and probiotics, will also have a relatively high effect size. In general, the effect size of nutritional intervention is known to be inherently low, and a relatively high effect size can only be expected when starting at (very) young age. The cumulative effect size of a nutritional intervention, here defined as the total effect starting from the age one starts with intervention till the end of life, will be inversely proportional to the age one starts with the intervention [Adapted from personal communication with Prof. R.J. Brummer, School of Medical Sciences, Örebro University, Sweden]. The later one starts, the smaller the cumulative effect size can be expected. As interventions explicitly targeting the gut microbiota may result in permanent alterations before the age of three, an even higher cumulative effect size may be expected during this time window.

**FIGURE 1:**
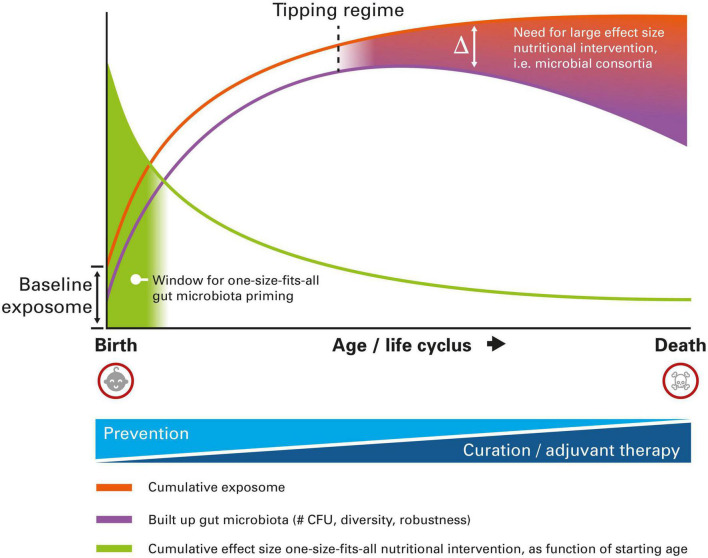
Schematic representation of the different nutritional intervention modalities targeting the gut microbiota to reduce the burden of autoimmune disease, as a function of age. At very early age, one-size-fits-all intervention is still possible, whereas at older age a more personalized approach is needed, due to higher interindividual variation imposed by the cumulative exposome. For details, see text.

The gut microbiota composition changes throughout life (29). At very young age, it is mainly determined by bifidobacteria and a low diversity (30). During adulthood, a robust and diverse microbiota is being observed, whereas a lower diversity gut microbiota having a more pro-inflammatory character (inflammageing) is being observed at old age (31). Autoimmune disorders may have already become phenotypic during ageing, which are also associated to changes in the gut microbiota. On top of that, the gut microbiota composition is being influenced by all kinds of personalized exposome factors. The inherently large interindividual variation at older age most likely calls for, in case of complex pathophysiology which is the case for autoimmune disorders, a more personalized and tailor-made intervention. Effective gut microbiota intervention will require a more and more personalized approach, fitting with the unique microbial composition of the individual. One-size-fits-all probiotic products could certainly still be of use at older age, albeit with a limited effect size, especially when the start of consumption begins at relatively older age and for indications with complex pathophysiology involving complex gut microbial changes. The aforementioned discussion is graphically summarized in Figure 1.

The call for a personalized gut microbiota intervention at older age is illustrated by the results that are being obtained using fecal microbiota transplantation. A recent meta-analysis clearly demonstrated the potential of FMT for autoimmune disorders, like Type-1 diabetes and inflammatory bowel disease (32). However, although FMT provides a patient with a completely new gut microbial ecosystem, it’s efficacy seems still to be determined by baseline characteristics of the patient. For example, in a trial in which it was tried to improve insulin sensitivity for patients suffering from metabolic syndrome, only 50% of the patients responded positively on the transplant (33). It was shown that the baseline diversity of the patient’s gut microbiota was the determinant for being either a responder or non-responder. Hence, also the supplementation of large microbial consortia (of which FMT is the “in extremo” case) calls for a personalized approach.

As FTM is a very invasive technique, suffering also from inherent possible safety issues (34), one would like to search for saver and more patient friendly alternatives. Ideally, one would like to supply nutrition containing the consortia needed to restore the microbiota elements that are malfunctioning or even absent because of the presence of (autoimmune) disease. Hence, knowledge on the microbial signatures that are associated with (autoimmune) disease are urgently needed. These signatures will most likely consist of keystone taxa (35) and microbial ecosystems dedicated to a specific functionality, also called microbial guilds (36). Supplementation of such multi-taxa probiotic preparations dedicated to restore keystone and/or guild features are still one of the future perspectives of healthcare, but first estimates on the ecological dimensions of such preparations have already been published (37). Current multi-taxa probiotic preparations that are on the market are not designed with this underlying rationale, and it was recently shown that the efficacy of multi preparations in general do not outperform that of single-strain preparations yet (38).

Summarizing, gut microbial intervention using nutrition does show promising potential to lower the burden of autoimmune type indications. Early life intervention is expected to exert the highest effect sizes, with inherently less need for a personalized approach due to the relatively low interindividual variation. With increasing age, the call for a more personalized intervention gets stronger and stronger, as the gut microbiota evolved in a unique way, determined by the personalized cumulative exposome. Challenges lie in the identification of microbial blueprints (or, more likely, ranges of blueprints) that are connected to autoimmune disorders. These blueprints will consists of keystone taxa and microbial guilds. The elucidation of these elements opens the possibility to, ultimately, supply these microbial consortia through personalized nutrition in order to reduce the burden of autoimmune disease. Currently, an unambiguous analysis of the gut microbiota composition is hampered by the notion that there is variation in outcomes between different methods used (39), effectively hindering the identification of microbial blueprints for autoimmune disorders as well. As such, one cannot identify the “real” microbial blueprint of a person (or range of blueprints, arising from possible interindividual variation). These differences in outcome are recognized to be caused by a variety of factors, like differences in sampling methods (40), sequencing methods (41), post-sequencing software tools, and microbial gene databases (42). Hence, for accurate identification of possible microbial blueprints it is a prerequisite that the differences between all these techniques and tools are known and, if possible, eliminated.

Next to the aforementioned challenges regarding the correction identification of the gut microbial content itself, large differences depending on factors like living arrangement and geographical location will remain. As such, it may be possible that not only the administration of microbial consortia has to be optimized regarding the target gut microbiota composition, but that a one-size-fits all interventional modality also has to be optimized for the population living in the target geographical location with associated living arrangement (urban or rural).

## Data availability statement

The original contributions presented in this study are included in the article/supplementary material, further inquiries can be directed to the corresponding author.

## Author contributions

The author confirms being the sole contributor of this work and has approved it for publication.
